# Splenic Artery Embolization as a Primary Treatment for Hereditary Spherocytosis: A Case Report

**DOI:** 10.7759/cureus.79269

**Published:** 2025-02-19

**Authors:** Habiba A Mahamat, Shoroq M Alamin, Amani Alrawi, Omar A Al Mohammad, Ali Alsaadi, Saud Balila

**Affiliations:** 1 Faculty of Medicine, International University of Africa, Khartoum, SDN; 2 College of Medicine, Sulaiman Alrajhi University, Al-Qassim, SAU; 3 Medicine and Surgery, Elrazi University, Khartoum, SDN; 4 Interventional Radiology, Ministry of Health Holdings, Madinah, SAU; 5 Hematology, Ministry of Health Holdings, Madinah, SAU

**Keywords:** chronic anemia, hereditary spherocytosis, minimally invasive procedure, splenic artery embolization, total splenectomy

## Abstract

The primary management of hereditary spherocytosis (HS) typically involves splenectomy to prevent hemolytic crises. However, splenic artery embolization (SAE) has emerged as a promising minimally invasive alternative. We report a case of a woman in her mid-20s with severe HS who presented with chronic fatigue, anemia resistant to iron supplementation, and splenomegaly. The diagnosis was confirmed through clinical findings, osmotic fragility test, and laboratory results. A partial embolization of the lower two-thirds of the spleen was performed while preserving the function of the upper third. Post-procedure, significant improvements were observed in hemoglobin levels (rising from 7.7 g/dL to 11.3 g/dL), hematocrit, and bilirubin levels, with no major complications reported. This case highlights that partial selective splenic artery embolization (PSE) is a safe and effective non-surgical procedure that can be considered as a potential alternative to splenectomy in the management of hereditary spherocytosis. Further studies are warranted to establish its long-term efficacy.

## Introduction

Hereditary spherocytosis (HS) is the most common congenital hemolytic anemia with a prevalence of one in 2,000 individuals of northern European ancestry. However, its prevalence in people of other ethnic backgrounds is yet to be known [1]. Its clinical presentation varies based on disease severity and genetic mutation type, encompassing manifestations from anemia and jaundice to splenomegaly, fatigability, pallor, and abdominal pain. The diagnosis of HS is the result of collaboration between history, clinical presentation, and laboratory results. A positive diagnosis can be confirmed through specialized tests such as osmotic fragility, eosin-5-maleimide (EMA), or acidified glycerol lysis test (AGLT). Managing HS includes general supportive measures, such as folic acid supplementation, while the primary treatment is total splenectomy to prevent hemolytic crisis. Alternatively, a minimally invasive management modality through angiographic embolization of the splenic artery is an effective method for controlling hemolysis, increasing hemoglobin levels, and reducing reticulocyte and bilirubin counts. This minimally invasive approach offers rapid clinical improvements; however, potential risks such as splenic infarction and infection require careful patient selection [2-4]. We report a case of a woman with severe hereditary spherocytosis that was primarily and effectively treated with partial selective splenic artery embolization (PSE) in King Salman Bin Abdulaziz Medical City, Medina, Saudi Arabia, which was the first case ever to undergo this procedure in our center.

## Case presentation

A 27-year-old woman with a known history of hypothyroidism, for which she was not on medication, and gallstones presented to King Salman Hospital with acute, severe, generalized abdominal pain that had been progressively worsening over the past few days. The patient also reported chronic headaches and easy fatigability, which had been present since she was diagnosed with iron deficiency anemia in 2015, negatively impacting her quality of life. She denied any fever, vomiting, changes in bowel habits, or weight loss. Her anemia had been refractory to a full three-month course of oral iron therapy. Her sister underwent cholecystectomy at the age of 22 due to gallstones, and there is a family history of unexplained splenomegaly. She has no history of blood transfusion or previous surgeries. On general examination, she was vitally stable but appeared icteric and pale. Abdominal examination revealed generalized tenderness, especially in the left upper quadrant, without guarding or rigidity, and with positive bowel sounds. Other systems examinations were unremarkable. An ultrasound showed an enlarged spleen reaching a maximum size of 22 cm and no suspicious focal lesions.

Recent laboratory results, including a complete blood count, revealed the following abnormalities: white blood cell count of 15.27 × 10^9^/L, hemoglobin of 7.7 g/dL, and slightly elevated bilirubin levels of 25 µmol/L, while liver function tests (LFTs) were within normal limits (Table 1). We ruled out other potential causes of anemia by checking serum ferritin levels, iron studies, and reticulocyte counts, all of which were normal. A peripheral blood smear showed spherocytosis, the direct Coombs test was negative, and the osmotic fragility test showed increased erythrocyte fragility. Based on the clinical features and confirmatory tests, a diagnosis of hereditary spherocytosis was established, and immediate folic acid supplementation was started. Consultation with interventional radiology was conducted for splenic artery embolization after the patient refused splenectomy.

**Table 1:** Laboratory results at presentation and six weeks and six months post-procedure Hb: hemoglobin, WBC: white blood cell, RBC: red blood cell, HCT: hematocrit, MCV: mean corpuscular volume, MCH: mean corpuscular hemoglobin, MCHC: mean corpuscular hemoglobin concentration, RDW: red cell distribution width, AST: aspartate aminotransferase, GOT: glutamic-oxaloacetic transaminase, ALT: alanine aminotransferase, GPT: glutamic pyruvic transaminase, BUN: blood urea nitrogen

Parameters	At presentation	6 weeks after	6 months after	Normal range
Hb (g/L)	7.7	9.9	11.3	12-15
WBC (×10^9^/L)	9.92	12.3	11	4-10
RBC (×10^12^/L)	3.09	4.5	5	3.8-4.8
HCT (g/dL)	24.8	30.7	34.7	36-46
MCV (fL)	80.3	68.1	69.4	83-101
MCH (pg)	24.9	22	22.6	27-32
MCHC (g/dL)	31	32.2	32.5	31.5-34.5
RDW (%)	19.5	22.9	21.3	11.6-14
Platelets (×10^9^/L)	376	1,078	761	150-450
Direct bilirubin (μmol/L)	4.3	3.35	3.70	0-3
Total bilirubin (μmol/L)	25	12.7	15	3-17
AST (GOT) (U/L)	8	13	14	15-37
ALT (GPT) (U/L)	4	22	21	14-59
Alkaline phosphatase (U/L)	75	92	N/A	46/116
Creatinine (μmol/L)	47	62	49	49-90
BUN (mmol/L)	1.2	1.4	N/A	2.5-6.4

**Figure 1:**
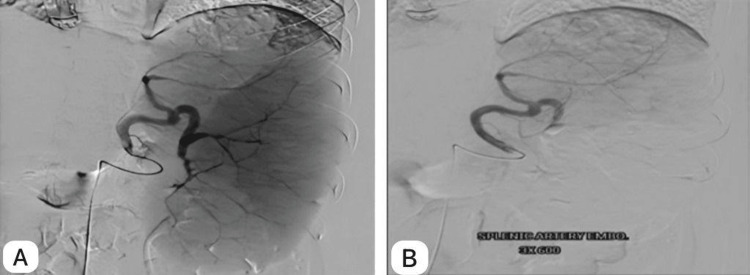
Splenic artery embolization A: Splenic artery angiogram showing the splenic enlargement and outline of the vascular anatomy. B: Post-embolization with occlusion of the middle and lower third artery preserving the upper branch.

Through right femoral artery access, a catheter was inserted, and selective catheterization of the celiac and then splenic artery was performed. A splenic artery angiogram revealed an enlarged spleen and highlighted the splenic vascular anatomy. Embolization of the lower two-thirds of the spleen was performed utilizing polyvinyl alcohol (PVA) (particle size: 300-500 microns) with successful occlusion of the middle and lower arterial supply of the spleen, preserving the upper third function (Figure 1). Targeting the lower two-thirds helped prevent pleural irritation while preserving part of the spleen to retain its functional benefits. The goal was to reduce splenic size without compromising its essential functions.

**Figure 2:**
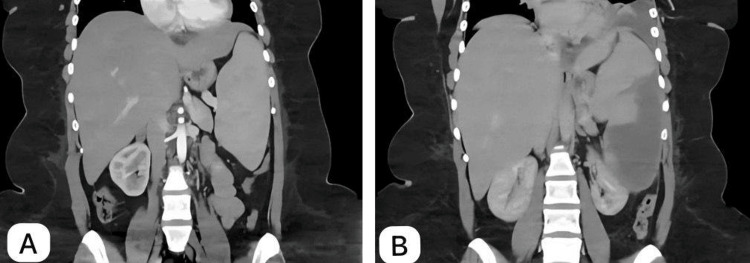
Abdominal CT with contrast A: Pre-embolization abdominal CT: A coronal CT image of the abdomen obtained before embolization shows an enlarged spleen due to hereditary spherocytosis, with no other significant findings. B: Post-embolization abdominal CT: A coronal CT image of the abdomen taken three months after embolization demonstrates a significant splenic infarction affecting the lower two-thirds of the spleen, while the upper third remains preserved. Mild left pleural effusion is also noted, likely representing a post-procedural reaction. CT: computed tomography

A follow-up contrast-enhanced abdominopelvic CT scan was conducted three months after the procedure, which showed middle and lower zone splenic infarct with mild left-side pleural reaction (Figure 2).

## Discussion

Hereditary spherocytosis (HS) is an autosomal dominant hematologic disorder characterized by anomalies in the structural proteins of erythrocytes, resulting in their distinctive spherical morphology rather than the typical biconcave disc shape. These anomalies arise from mutations affecting critical cytoskeletal proteins involved in maintaining the integrity and flexibility of the erythrocyte membrane, notably spectrin, ankyrin, band 3 protein, or protein [4]. Consequently, weakened interactions between the lipid bilayer and the underlying cytoskeleton compromise membrane stability, leading to decreased surface area and the formation of spherical red cells known as spherocytes. The spleen, recognizing these aberrant erythrocytes as defective, prematurely removes them from the circulation, precipitating chronic hemolytic anemia. Clinical manifestations typically include anemia-related symptoms such as fatigability, weakness, and pallor, alongside jaundice stemming from elevated bilirubin levels due to accelerated erythrocyte breakdown. Additionally, splenomegaly frequently ensues as the spleen intensifies its efforts to clear the abnormal erythrocytes, potentially causing abdominal discomfort or early satiety. Furthermore, excessive erythrocyte breakdown can cause bilirubin gallstone formation, contributing to abdominal pain and complications [1].

The diagnosis of HS employs various methodologies, including examination of a peripheral blood smear, which typically reveals an augmented number of spherocytes and variable degrees of anemia. The osmotic fragility test, evaluating erythrocyte susceptibility to lysis in hypotonic solutions, and analysis by flow cytometry that measures the fluorescence intensity of labeled intact red cells serve to corroborate the diagnosis. Moreover, genetic analysis can identify specific mutations associated with HS, shedding light on the inheritance pattern, predominantly autosomal dominant, although autosomal recessive and de novo mutations can also manifest [5].

Regarding treatment, there is currently no specific therapy available to correct the cytoskeletal membrane defects of erythrocytes in HS. However, splenectomy remains the primary therapeutic option for patients presenting with moderate to severe symptoms. Additionally, symptomatic control measures such as lifelong folic acid supplementation are recommended to prevent megaloblastic crises due to chronic hemolysis [3,6]. This surgical intervention alleviates the symptoms that are associated with chronic hemolysis, including anemia, splenomegaly, recurrent gallstones, and, in some cases, skeletal changes resulting from extramedullary hemolysis [2,7-9]. The decision to proceed with splenectomy hinges upon several factors, including the patient's age, the severity of anemia, and a thorough discussion regarding the potential long-term complications. While splenectomy has demonstrated efficacy in improving anemia, it carries a lifelong risk of potentially fatal sepsis or meningitis due to decreased antibody titers against pneumococcal serotypes and a diminished response to pneumococcal immunization [10]. Moreover, the increased risk of stroke and myocardial infarction post-splenectomy is attributed to elevated hemoglobin levels as well as reactive thrombocytosis in splenectomized individuals compared to unaffected family members. Pulmonary hypertension appears to be more prevalent in individuals undergoing splenectomy, highlighting the importance of careful patient selection and risk assessment [11]. Partial splenectomy has emerged as an alternative in specific cases, offering a reduction in encapsulated bacterial infection risk; however, it may result in persistent mild chronic hemolysis and an increased likelihood of gallbladder stone formation [12]. Additionally, cases of acute severe anemia and splenomegaly necessitating splenic remnant removal post-partial splenectomy have been documented [2]. Laparoscopic surgery is often preferred for managing hematologic disorders associated with splenomegaly, although recent evidence suggests comparable perioperative outcomes between laparoscopic and open methods, particularly in individuals with moderate to severe splenomegaly [9,13]. Furthermore, embolic therapy, initially described in 1971 for hypersplenism, has evolved into a viable treatment option for various diseases, including HS [13].

Partial splenic artery embolization has emerged as a minimally invasive alternative to splenectomy, demonstrating safety and efficacy in improving hematologic parameters while preserving residual splenic function and minimizing postoperative complications. Super-selective partial splenic embolization, particularly in pediatric cases, offers precise control over the degree of embolization, optimizing therapeutic outcomes while minimizing collateral damage. In a study conducted by Takahashi et al., it was concluded that splenic artery embolizations performed prior to laparoscopic splenectomies were not only free of complications but also successful in reducing intraoperative blood loss [14]. They asserted that splenic artery embolization is a safe and beneficial adjuvant procedure performed before elective laparoscopic splenectomy in children. In contrast to their approach, our case report focused solely on splenic artery embolization without subsequent splenectomy. This was due to the patient's refusal of splenectomy. Despite differences in treatment strategy, our results were both successful and promising. After a six-month follow-up, the patient demonstrated notable clinical improvement, supported by laboratory and radiological progress. Hemoglobin level raised from 7.7 g/L to 9.9 g/L, then to 11.3 g/L. Hematocrit increased from 24.8 g/dL to 30.7 g/dL, then to 34.7 g/dL. WBC initially raised from 9.92 × 10^9^/L to 12.3 × 10^9^/L and then stabilized at 11 × 10^9^/L. Platelet counts, which initially increased from 376 × 10^9^/L to 1,078 × 10^9^/L, eventually settled at 761 × 10^9^/L. Total bilirubin level dropped from 25 µmol/L to 15 µmol/L within six weeks and then further to within the normal range after six months (Table 1). Additionally, a marked thrombocytosis, with levels reaching up to 1,320 × 10^3^/µL, was observed. Other studies have also reported similar increases in thrombocytosis in patients with HS following splenic artery embolization [2]. Although splenectomy has been widely recognized as an effective management strategy for HS, with positive outcomes reported in numerous literature reviews, our case report underscores the efficacy of splenic artery embolization. However, additional observations, such as clinical assessment of anemia symptoms, serial hemoglobin levels and reticulocyte counts, and abdominal CT scan are necessary to establish the indications for this treatment method and to evaluate its long-term outcomes [14].

## Conclusions

This case was reported to prove that PSE is a safe and effective non-surgical procedure that can be considered as a potential alternative to splenectomy in the management of hereditary spherocytosis.
